# Complicated Dengue Fever and Its Treatment Dilemmas: A Single-Center Experience in Sri Lanka

**DOI:** 10.1155/2021/8854282

**Published:** 2021-01-13

**Authors:** D. K. Jayaweera, S. Subasinghe, R. F. De Silva, W. A. H. P. Sanjeewa, K. P. Jayawickreme

**Affiliations:** Registrar in Medicine Ward 17 Sri Jayewardenepura General Hospital Sri Lanka, Sri Jayawardenepura Kotte, Sri Lanka

## Abstract

Dengue is an arboviral infection that affects humanscausing significant morbidity and mortality in tropical countries. Our first patient who had diabetes presented with shock and was managed as dengue hemorrhagic fever with superadded sepsis which required noradrenalin plus broad-spectrum intravenous antibiotics. The second patient developed severe bradycardia during the ascending limb of the critical phase with hemodynamic stability, which recovered on discharge. Third patient presented with severe and rapid leaking; we used intravenous albumin as an alternative colloid with good outcome. The fourth patient was a pregnant mother at term, and she went into spontaneous labor during the latter half of the critical phase. The fifth patient developed dengue hemorrhagic fever complicated with probable haemophagocytic lymphohistiocytosis. She was treated with intravenous steroids andimmunoglobulin, yet succumbed on day 7. *Conclusion*. Dengue is an extremely challenging and dynamic disease, which can lead to many unusual complications.A high index of suspicion is key to diagnose and treat promptly.

## 1. Introduction

Dengue fever is the most prevalent arboviral infection that affects humans with significant mortality. It has four serotypes that demonstrate various clinicopathologic manifestations in the human body, leading to different presentations and complications with unpleasant sequelae. Management of dengue fever in Sri Lanka is done according to the national guidelines published by the Ministry of Health in 2012 that was updated in 2018. However, atypical presentations and unusual complications fall beyond the scope of current guidelines that can push the clinician into unfamiliar terrain. This case series describes five unusual complications of dengue fever and different management strategies adopted at a single center in Sri Lanka. All five patients in this case series had serologically confirmed dengue fever and presented to Sri Jayewardenepura General Hospital (SJGH) in the latter half of 2019. They were all females aged between 23 and 65 years.

### 1.1. Case 1

A 53-year-old diabetic patient presented with fever for five days. She had arthralgia and myalgia with no focal symptoms in keeping with a viral infection. Examination revealed right hypochondrial tenderness and clinical evidence of leaking. Her peripheries were warm, and she was haemodynamicaly stable. On admission, her hematologic indices showed leucopenia and thrombocytopenia (white blood cell count (WBC) of 3300/*μ*l, platelet count of 29000/mL, and packed cell volume (PCV) of 38%). She had positive dengue IgM antibodies on day 5. Her serum creatinine was 69 *μ*mol/L (normal), and her liver function tests and serum electrolytes were within normal range. Initial ultrasound scan of the abdomenshowed free fluid in the hepatorenal pouch.

She was considered to be in the ascending limb of the critical phase and managed with intravenous normal saline 50 ml and oral fluid 50 ml per hour with dengue critical-phase monitoring. Her blood sugar on admission was 183 mg/dl. It was controlled with regular subcutaneous soluble insulin injections.

Six hours later, her blood pressure dropped to 77/55 mm/Hg without tachycardia. Surprisingly, her pulse was bounding and CRFT (capillary refill time) was <2 seconds. She had no fever or chest pain. Her urine output maintained adequately probably owing to hyperglycemia.

Initial fluid resuscitation was carried out with two crystalloid boluses of normal saline at 5 ml/kg given rapidly followed by intravenous dextran 5 ml/kg over 30 minutes. Since the PCV drop was steep, we transfused blood (5 ml/kg/hour). Her ECG was normal without conduction blocks. Troponin I was normal. Her serum electrolytes including ionized calcium was within normal limits (Ca^2+^: 1.1 mmol/). The C-reactive protein (CRP) level was elevated at 40 ng/ml. 2D echo showed EF 60% without evidence of myocarditis. Following fluid resuscitation, she had persistent low blood pressure with good pulse pressure without tachycardia.

We suspected superadded sepsis with dengue hemorrhagic fever and started her on inotropic support with intravenous noradrenaline. Due to her underlying plasma leakage, we restricted initial fluid resuscitation to the minimal volume to prevent fluid overload. We started intravenous meropenem 1 g 8 hourly after taking blood and urine for culture. Rest of the critical phase was managed with intravenous crystalloid and oral fluids accordingly. She recovered completely on day 7 and discharged on day 8.

### 1.2. Case 2

A 34-year-old previously healthy woman presented on the second day of fever. Her NS1 antigen test was positive. She was managed as dengue hemorrhagic fever because she had sonographic evidence of leakage. During the ascending limb of the critical period, she developed sinus bradycardia with the lowest heart rate of 37 beats per min (bpm). She remained haemodynamically stable.

Her troponin I was negative. 2D echocardiogram was normal, and ejection fraction was >60%. Her thyroid functions were TSH: 3.084 mIU/L and T4: 1.21 mIU/L.

We started her on oral orciprenaline 5 mg bd after discussing with the consultant electrophysiologist. Later, the dose was doubled to maintain her heart rate above 60bpm. Once she recovered from dengue hemorrhagic fever, her heart rate picked up and orciprenalin dose was gradually tailed off over 48 hours. The critical period was otherwise uneventful. Subsequent 24-hour holter monitoring done two weeks after recovery was normal.

We concluded this to be a case ofdengue fever-associated sinus node dysfunction.

### 1.3. Case 3

A 23-year-old previously healthy woman presented with typical symptoms of dengue fever on day 2 with positive NS1 antigen test. On admission, her WBC was 2900/*μ*l with a platelet count of 158000/*μ*l. On day 3, her platelets dropped precipitously to 29,000/*μ*l with evidence of plasma leaking on ultrasound scan. Dengue fluid management was done according to national guidelines.

During the middle of the dengue critical phase (20 hours), she developed tachycardia (heart rate was 127 beats/min) with a narrow pulse pressure of 27 mmHg. Her urine output was reduced to <0.5 ml/kg/hour for 3 hours. Her hematocrit had risen up to 57% (baseline 45%). She had clinical features of fluid overload with bilateral moderate pleural effusions. At this point, we had already given the total dextran quota for 24 hours (3 × 10 ml/kg). We had given total fluid quota of 3550 ml.

Her serum albumin level was 1.7 g/dL. We took expert opinion and gave intravenous salt poor albumin 100 ml over one hour. Her parameters including tachycardia, narrow pulse pressure, and urine output improved after the albumin bolus.

We repeated albumin boluses of 100 ml at 25^th^ and 39^th^ hour of the critical phase. We reduced oral intake 25 ml to 50 ml per hour in between albumin boluses to prevent further fluid overload. We did not use any intravascular crystalloids during this period. The patient recovered and was discharged safely on day 8.

### 1.4. Case 4

A 34 year-old woman during her second pregnancy presented at 37 weeks of POA with fever on day 3 and positive NS1 antigen test. She developed progressive thrombocytopenia below 100,000/*μ*l and managed as dengue hemorrhagic fever with hourly monitoring. However, she did not develop ultrasound evidence of plasma leakage. At the latter stage of the critical phase, she went into spontaneous labor. At this point, her platelet count was 47000/*μ*l with one rising platelet count (lowest platelet was 43000/*μ*L). Since she had past LSCS (lower segment caesarian section), obstetrics team decided to proceed with emergency LSCS for the delivery. We faced a management dilemma at this point because still she had low platelet count.

A multidisciplinary discussion was done involving the hematology, transfusion medicine, and obstetric teams. Final decision was to proceed with immediate lower segment caesarian section.

A manual platelet count was done just before surgery. It was 54,000/*μ*LHer immature platelel fraction was 21%. No platelet transfusion was done. Blood and platelets were preserved. She did not develop any major bleeding and was discharged with hernewborn safely on day 8.

### 1.5. Case 5

A 27-year-old woman presented with NS1 antigen positive dengue fever on day 3. She had ultrasound evidence of leaking on admission and was managed as dengue hemorrhagic fever. Unusually, her fever spikes persisted along with bicytopenia (platelet 15000/*μ*l, Hb 8 g/dl) well past day 6.

She had significant elevated hepatic transaminases (AST 12841 U/L and ALT 3034 U/L). Screening for acute viral hepatitis was negative. Serum ferritin was 136,459 ng/mL. Her blood picture showed dengue hemorrhagic fever with superadded bacterial infection. USS abdomen detected free fluid in pelvis without hepatomegaly or splenomegaly. Her cardiac function was good with normal 2D echo and an ejection fraction of 60%. ROTEM (rotational thromboelastography) showed severe coagulation derangement. Her CRP was 13 and blood cultures remained negative.We transfused her fresh frozen plasma, platelet, and cryoprecipitate and started her on intravenous Ceftriaxone. Clinical diagnosis of hemophagocytic lymphohistiocytosis was considered. Bone marrow aspiration was not done because the patient had high risk of bleeding and she was haemodynamically unstable. We treated her with IV methylprednisolone 1 g daily and IV immunoglobulin (0.4 g/kg/daily)for one day. However, she developed multiorgan failure with acute kidney injury. She succumbed on day 7.

## 2. Discussion

Dengue is a dynamic disease with variable presentations and an array of direct and indirect complications. Management of dengue fever in Sri Lanka is based on national dengue guidelines. It relies on prompt identification of plasma leakage or the onset of the critical phase, meticulous monitoring of parameters, fluid management and accurate recognition of the convalescent phase/fluid reabsorption. However, the sheer diversity of the clinico-pathologic outcomes of dengue fever underscores the importance of clinical experience to manage these clinical roadblocks. The above five case scenarios describe unusual complications of dengue fever and different management strategies applied for the complications.

Dengue hemorrhagic fever has three main phases. Febrile phase, critical phase, and recovery phase [1]. The febrile phase usually lasts for 2 to 7 days. Mild leucopenia (white blood cells <5000/*μ*L) and thrombocytopenia (<150000/*μ*l) can be observed during this period. Some patients will go to the critical phase or leaking phase with the evidence of plasma leaking. This usually lasts for 24 to 48 hrs. Fluid leakage will be selective and limited to pleural and peritoneal spaces. Rising hematocrit, low albumin, and low total cholesterol are other signs of fluid leaking.

During the recovery phase, patients' general wellbeing is improved and leaked fluid will be reabsorbed to the intravascular system [1].

The first case is of dengue hemorrhagic fever and persistent shock not responding to guideline-based fluid resuscitation. The presence of bounding pulse and preserved pulse pressure with a low diastolic blood pressure is not in keeping with plasma leakage seen in dengue fever [1]. Although no obvious focus was apparent, presence of underlying immunosuppression in the background of poorly controlled diabetes made us suspect secondary bacterial sepsis leading to shock. She had no tachycardia probably secondary to dengue-associated sinoatrial node dysfunction [2, 3], and her urine output remained normal probably secondary to hyperglycemia-associated diuresis. Anaphylaxis is also unlikely in the absence of any trigger, urticaria, wheezing, or angioedema and good response to inotropic support.

Low blood pressure due to sepsis requires a large volume fluid resuscitation (30 ml/kg in first hour) with crystalloid boluses [4]. It is detrimental in dengue patients with plasma leakage as subsequent fluid overload increases mortality. We used early vasopressors and antibiotics other than the usual controlled dengue fluid management. Clear differentiation of dengue vs. septic shock is always challenging in tropical countries [5, 6]. Missed diagnosis would yield worse outcome considering the significant differences in fluid management [6].

Dengue-related cardiac dysfunctions are quite uncommon [3]. Out of the dengue-related cardiac dysfunctions, the most commonly reported is sinus bradyarrhythmia [2]. Usually, this recovers spontaneously [7], although rare instances requiring temporary pacing has been reported [7]. Our second patient developed sinus bradycardia during the dengue critical phase with heart rate dropping below 40 bpm. Although she was haemodynamically stable considering the imminent risk of plasma leakage and low blood pressure, orciprenaline was started after discussing with the consultant cardiac electrophysiologist. Dengue-related bradycardia is usually treated with intravenous atropine or oral orciprenaline [2].

Rapid and prolonged plasma leakage is a stressful situation to the clinician. It is impossible to limit to the allocated fluid quota if the patient is hemodynamically unstable. On the other hand, overtreatment with crystalloids leads to fluid overload which undermines perfusion. When the patient enters the descending limb of the critical phase, fluid which leaked before will spontaneously reabsorb to the circulation. Our third patient's leaking phase was quite prolonged. She had persistent tachycardia, rising hematocrit, and low urine output in keeping with severe plasma leakage. Plasma leakage is known to have low albumin [1]. However, its use as a colloid was recognized only in few case series, and it has not come into guidelines. Our third patient responded well with intravenous albumin. We administered multiple short boluses of intravenous albumin timed with clinical deterioration and managed to achieve adequate perfusion. Although she developed features of fluid overload with bilateral pleural effusions, saving her life was a victory. Use of albumin as a colloid in dengue shock is under investigation by a team in Indonesia, but results are yet to be published.

Dengue in pregnancy is challenging because of the altered physiology compared to a healthy nonpregnant woman. Our patient went into spontaneous labor during the descending limb of the dengue critical phase. Mode of delivery during the critical phase of dengue is decided in favor of the mother's life irrespective of the outcome of the baby [8]. Since she was in her second pregnancy with past LSCS, the preferred mode of delivery was LSCS. Performing caesarian section witha low platelet count is challenging. Platelet count of more than 50,000/*µ*L is the cutoff recommended for surgery [8]. We used immature platelet fraction as a surrogate marker of recovery of platelet in dengue. IPF of >10% predicted the recovery of platelet counts within 24 to 48 hours[9]. Our patient's IPF was 21% at the onset of labour. Other major complication of delivery in dengue is postpartum bleeding [8]. This could be due to low platelet, clotting derangement, and uterine atony [8,10].

Haemophagocytic lymphohistiocytosis (HLH) describes a clinical syndrome of hyperstimulation and ineffective immune response to infection or malignancy [11]. HLH is a recognized complication of dengue with high mortality [12]. Our last patient had continuous fever beyond 6 days with persistent bicytopenia (low white blood cells and platelets; lowest 1700/*μ*L and 12,000/*μ*L, respectively) which is not compatible with isolated dengue infection. Her blood cultures were negative and CRP was 13. She had evidence of hepatitis. Possibility of disseminated intravascular coagulation was excluded. Although she had only 3/5 of the HLH 2004 criteria, she had a very high ferritin level of >130,000 ng/ml.Allen et al. in their study demonstrated a high sensitivity and specificity for HLH in patients with ferritin level >10,000ng/ml, especially in those with new onset febrile illness without background medical history[13].H. Our working clinical diagnosis was HLH, and we treated her with intravenous immunoglobulin 0.4 g/kg daily and IV methylprednisolone 500 mg daily for 2 days. However, she succumbed following multiorgan dysfunction. Our patient fits into this group of extreme hyperferritinemia which is well associated with HLH. Diagnosis with tissue confirmation is usually not feasible with haemodynamic instability. Hence, a clinical diagnosis may have to be made to initiate treatment.

## 3. Conclusion

Dengue is an extremely challenging and dynamic disease, which can lead to many unusual complications. Some of these complications can be fatal and require high index of suspicion to diagnose and treat promptly.
